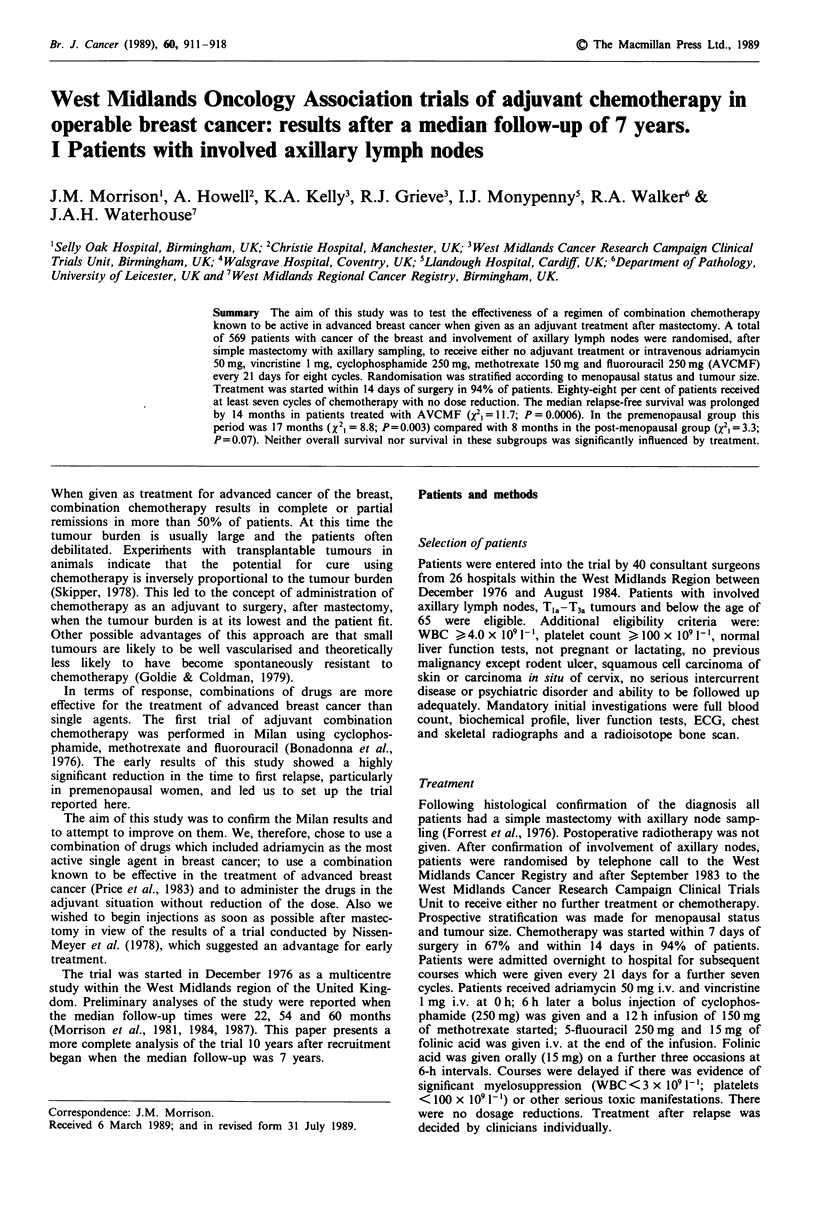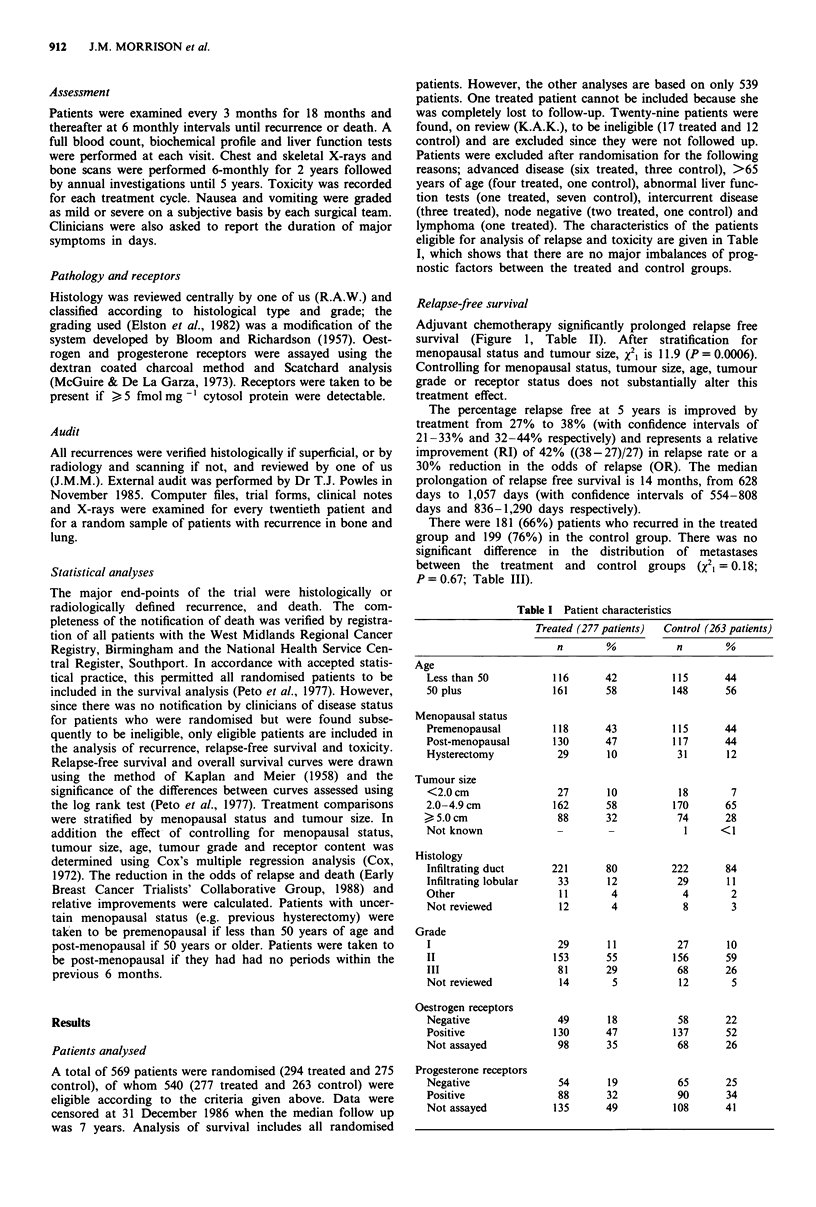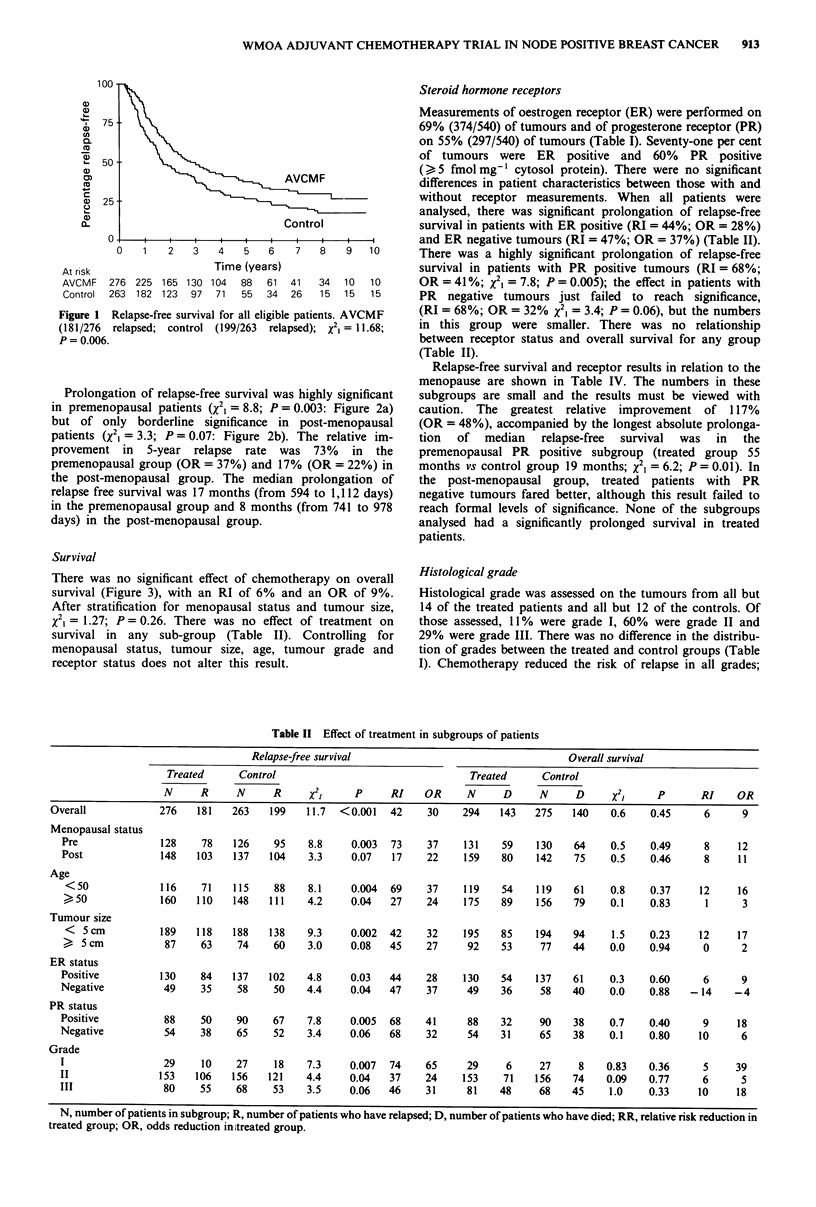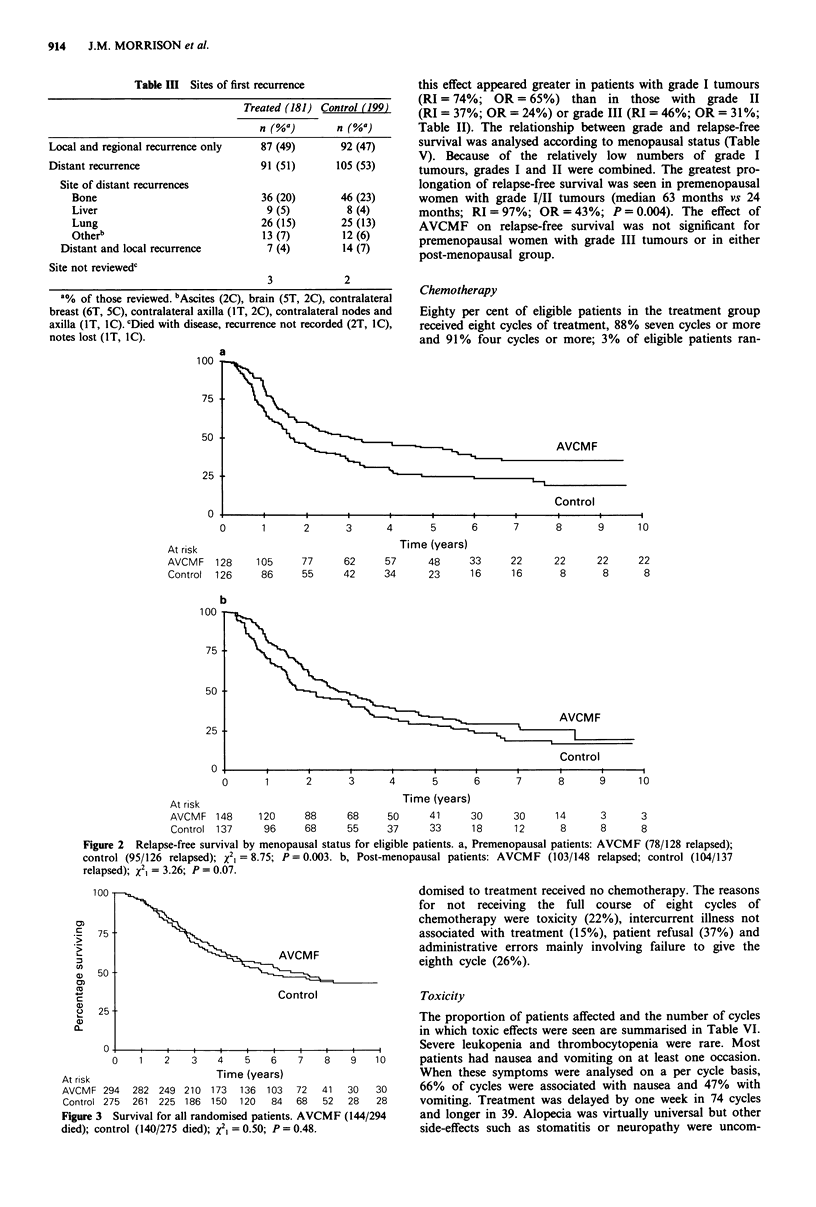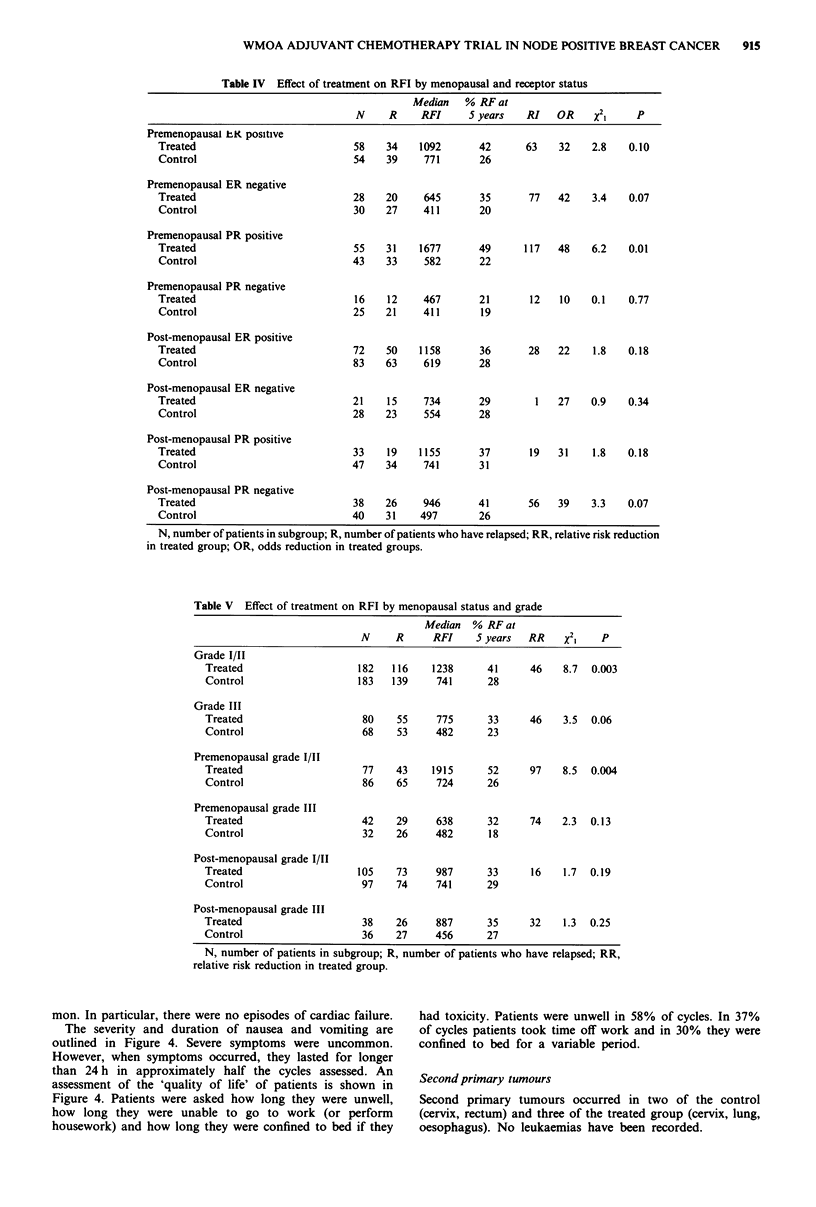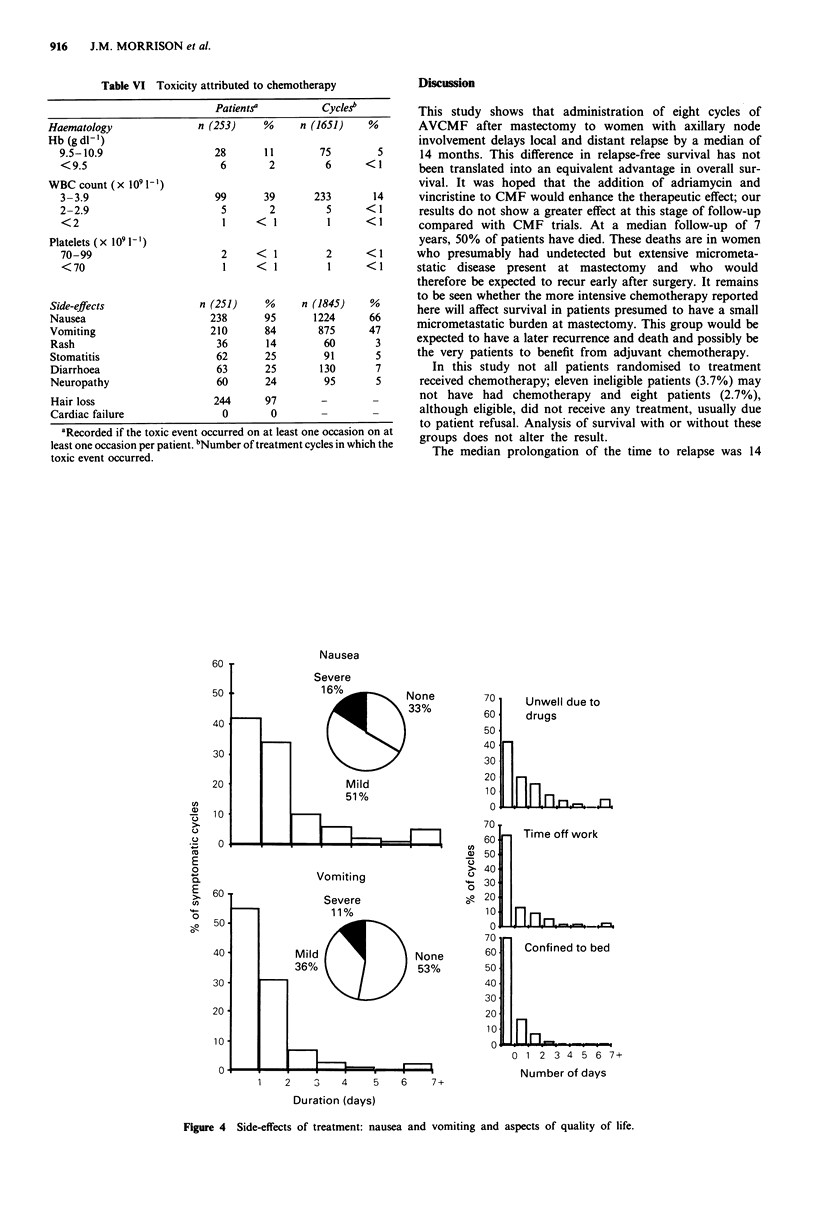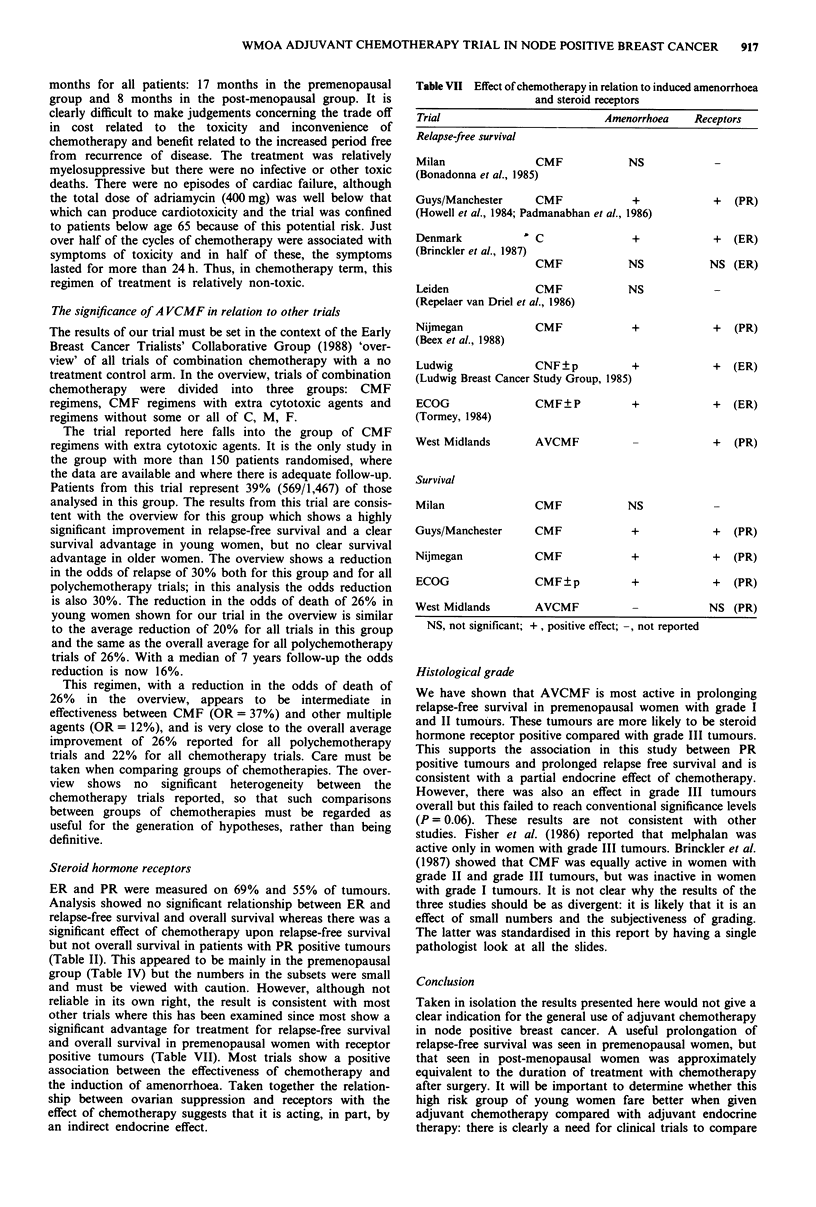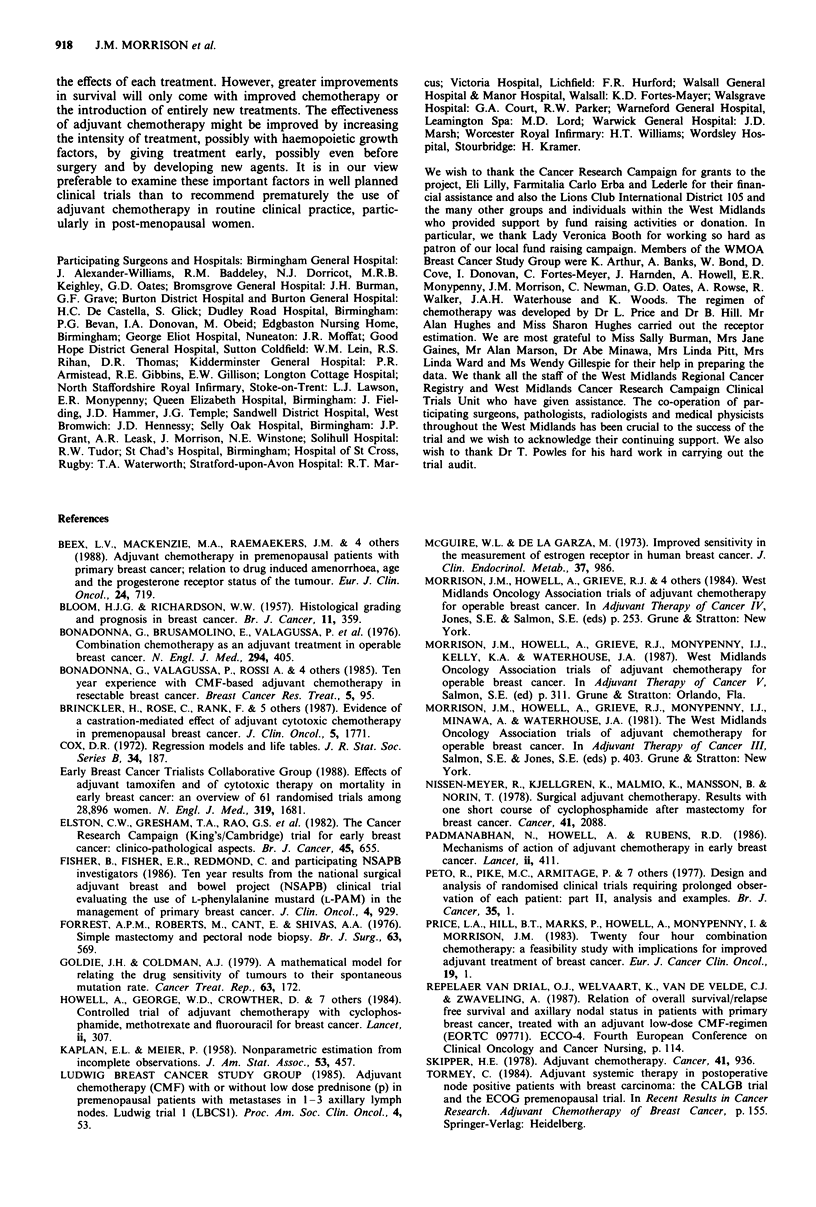# West Midlands Oncology Association trials of adjuvant chemotherapy in operable breast cancer: results after a median follow-up of 7 years. I. Patients with involved axillary lymph nodes.

**DOI:** 10.1038/bjc.1989.389

**Published:** 1989-12

**Authors:** J. M. Morrison, A. Howell, K. A. Kelly, R. J. Grieve, I. J. Monypenny, R. A. Walker, J. A. Waterhouse

**Affiliations:** Selly Oak Hospital, Birmingham, UK.

## Abstract

The aim of this study was to test the effectiveness of a regimen of combination chemotherapy known to be active in advanced breast cancer when given as an adjuvant treatment after mastectomy. A total of 569 patients with cancer of the breast and involvement of axillary lymph nodes were randomised, after simple mastectomy with axillary sampling, to receive either no adjuvant treatment or intravenous adriamycin 50 mg, vincristine 1 mg, cyclophosphamide 250 mg, methotrexate 150 mg and fluorouracil 250 mg (AVCMF) every 21 days for eight cycles. Randomisation was stratified according to menopausal status and tumour size. Treatment was started within 14 days of surgery in 94% of patients. Eighty-eight per cent of patients received at least seven cycles of chemotherapy with no dose reduction. The median relapse-free survival was prolonged by 14 months in patients treated with AVCMF (chi2 1 = 11.7; P = 0.0006). In the premenopausal group this period was 17 months (chi2 1 = 8.8; P = 0.003) compared with 8 months in the post-menopausal group (chi2 1 = 3.3; P = 0.07). Neither overall survival nor survival in these subgroups was significantly influenced by treatment.


Br. J. Cancer (1989), 60, 911–918 © The Macmillan Press Ltd., 1989

### West Midlands Oncology Association trials of adjuvant chemotherapy in operable breast cancer: results after a median follow-up of 7 years. I Patients with involved axillary lymph nodes

J.M. Morrison[1], A. Howell[2], K.A. Kelly[3], R.J. Grieve[3], I.J. Monypenny[5], R.A. Walker[6] & J.A.H. Waterhouse[7]

[1]*Selly Oak Hospital, Birmingham, UK;* [2]*Christie Hospital, Manchester, UK;* [3]*West Midlands Cancer Research Campaign Clinical Trials Unit, Birmingham, UK;* [4]*Walsgrave Hospital, Coventry, UK;* [5]*Llandough Hospital, Cardiff, UK;* [6]*Department of Pathology, University of Leicester, UK and* [7]*West Midlands Regional Cancer Registry, Birmingham, UK.*

**Summary** The aim of this study was to test the effectiveness of a regimen of combination chemotherapy known to be active in advanced breast cancer when given as an adjuvant treatment after mastectomy. A total of 569 patients with cancer of the breast and involvement of axillary lymph nodes were randomised, after simple mastectomy with axillary sampling, to receive either no adjuvant treatment or intravenous adriamycin 50 mg, vincristine 1 mg, cyclophosphamide 250 mg, methotrexate 150 mg and fluorouracil 250 mg (AVCMF) every 21 days for eight cycles. Randomisation was stratified according to menopausal status and tumour size. Treatment was started within 14 days of surgery in 94% of patients. Eighty-eight per cent of patients received at least seven cycles of chemotherapy with no dose reduction. The median relapse-free survival was prolonged by 14 months in patients treated with AVCMF ($\chi^2_1 = 11.7$; $P = 0.0006$). In the premenopausal group this period was 17 months ($\chi^2_1 = 8.8$; $P = 0.003$) compared with 8 months in the post-menopausal group ($\chi^2_1 = 3.3$; $P = 0.07$). Neither overall survival nor survival in these subgroups was significantly influenced by treatment.

When given as treatment for advanced cancer of the breast, combination chemotherapy results in complete or partial remissions in more than 50% of patients. At this time the tumour burden is usually large and the patients often debilitated. Experiments with transplantable tumours in animals indicate that the potential for cure using chemotherapy is inversely proportional to the tumour burden (Skipper, 1978). This led to the concept of administration of chemotherapy as an adjuvant to surgery, after mastectomy, when the tumour burden is at its lowest and the patient fit. Other possible advantages of this approach are that small tumours are likely to be well vascularised and theoretically less likely to have become spontaneously resistant to chemotherapy (Goldie & Coldman, 1979).

In terms of response, combinations of drugs are more effective for the treatment of advanced breast cancer than single agents. The first trial of adjuvant combination chemotherapy was performed in Milan using cyclophosphamide, methotrexate and fluorouracil (Bonadonna *et al.*, 1976). The early results of this study showed a highly significant reduction in the time to first relapse, particularly in premenopausal women, and led us to set up the trial reported here.

The aim of this study was to confirm the Milan results and to attempt to improve on them. We, therefore, chose to use a combination of drugs which included adriamycin as the most active single agent in breast cancer; to use a combination known to be effective in the treatment of advanced breast cancer (Price *et al.*, 1983) and to administer the drugs in the adjuvant situation without reduction of the dose. Also we wished to begin injections as soon as possible after mastectomy in view of the results of a trial conducted by Nissen-Meyer *et al.* (1978), which suggested an advantage for early treatment.

The trial was started in December 1976 as a multicentre study within the West Midlands region of the United Kingdom. Preliminary analyses of the study were reported when the median follow-up times were 22, 54 and 60 months (Morrison *et al.*, 1981, 1984, 1987). This paper presents a more complete analysis of the trial 10 years after recruitment began when the median follow-up was 7 years.

Correspondence: J.M. Morrison.
Received 6 March 1989; and in revised form 31 July 1989.

### Patients and methods

### *Selection of patients*

Patients were entered into the trial by 40 consultant surgeons from 26 hospitals within the West Midlands Region between December 1976 and August 1984. Patients with involved axillary lymph nodes, $T_{1a}$–$T_{3a}$ tumours and below the age of 65 were eligible. Additional eligibility criteria were: WBC $\geqslant 4.0 \times 10^9\,l^{-1}$, platelet count $\geqslant 100 \times 10^9\,l^{-1}$, normal liver function tests, not pregnant or lactating, no previous malignancy except rodent ulcer, squamous cell carcinoma of skin or carcinoma *in situ* of cervix, no serious intercurrent disease or psychiatric disorder and ability to be followed up adequately. Mandatory initial investigations were full blood count, biochemical profile, liver function tests, ECG, chest and skeletal radiographs and a radioisotope bone scan.

### *Treatment*

Following histological confirmation of the diagnosis all patients had a simple mastectomy with axillary node sampling (Forrest *et al.*, 1976). Postoperative radiotherapy was not given. After confirmation of involvement of axillary nodes, patients were randomised by telephone call to the West Midlands Cancer Registry and after September 1983 to the West Midlands Cancer Research Campaign Clinical Trials Unit to receive either no further treatment or chemotherapy. Prospective stratification was made for menopausal status and tumour size. Chemotherapy was started within 7 days of surgery in 67% and within 14 days in 94% of patients. Patients were admitted overnight to hospital for subsequent courses which were given every 21 days for a further seven cycles. Patients received adriamycin 50 mg i.v. and vincristine 1 mg i.v. at 0 h; 6 h later a bolus injection of cyclophosphamide (250 mg) was given and a 12 h infusion of 150 mg of methotrexate started; 5-fluouracil 250 mg and 15 mg of folinic acid was given i.v. at the end of the infusion. Folinic acid was given orally (15 mg) on a further three occasions at 6-h intervals. Courses were delayed if there was evidence of significant myelosuppression (WBC $< 3 \times 10^9\,l^{-1}$; platelets $< 100 \times 10^9\,l^{-1}$) or other serious toxic manifestations. There were no dosage reductions. Treatment after relapse was decided by clinicians individually.

*Assessment*

Patients were examined every 3 months for 18 months and thereafter at 6 monthly intervals until recurrence or death. A full blood count, biochemical profile and liver function tests were performed at each visit. Chest and skeletal X-rays and bone scans were performed 6-monthly for 2 years followed by annual investigations until 5 years. Toxicity was recorded for each treatment cycle. Nausea and vomiting were graded as mild or severe on a subjective basis by each surgical team. Clinicians were also asked to report the duration of major symptoms in days.

*Pathology and receptors*

Histology was reviewed centrally by one of us (R.A.W.) and classified according to histological type and grade; the grading used (Elston et al., 1982) was a modification of the system developed by Bloom and Richardson (1957). Oestrogen and progesterone receptors were assayed using the dextran coated charcoal method and Scatchard analysis (McGuire & De La Garza, 1973). Receptors were taken to be present if $\geqslant 5$ fmol mg$^{-1}$ cytosol protein were detectable.

*Audit*

All recurrences were verified histologically if superficial, or by radiology and scanning if not, and reviewed by one of us (J.M.M.). External audit was performed by Dr T.J. Powles in November 1985. Computer files, trial forms, clinical notes and X-rays were examined for every twentieth patient and for a random sample of patients with recurrence in bone and lung.

*Statistical analyses*

The major end-points of the trial were histologically or radiologically defined recurrence, and death. The completeness of the notification of death was verified by registration of all patients with the West Midlands Regional Cancer Registry, Birmingham and the National Health Service Central Register, Southport. In accordance with accepted statistical practice, this permitted all randomised patients to be included in the survival analysis (Peto et al., 1977). However, since there was no notification by clinicians of disease status for patients who were randomised but were found subsequently to be ineligible, only eligible patients are included in the analysis of recurrence, relapse-free survival and toxicity. Relapse-free survival and overall survival curves were drawn using the method of Kaplan and Meier (1958) and the significance of the differences between curves assessed using the log rank test (Peto et al., 1977). Treatment comparisons were stratified by menopausal status and tumour size. In addition the effect of controlling for menopausal status, tumour size, age, tumour grade and receptor content was determined using Cox's multiple regression analysis (Cox, 1972). The reduction in the odds of relapse and death (Early Breast Cancer Trialists' Collaborative Group, 1988) and relative improvements were calculated. Patients with uncertain menopausal status (e.g. previous hysterectomy) were taken to be premenopausal if less than 50 years of age and post-menopausal if 50 years or older. Patients were taken to be post-menopausal if they had had no periods within the previous 6 months.

### Results

*Patients analysed*

A total of 569 patients were randomised (294 treated and 275 control), of whom 540 (277 treated and 263 control) were eligible according to the criteria given above. Data were censored at 31 December 1986 when the median follow up was 7 years. Analysis of survival includes all randomised patients. However, the other analyses are based on only 539 patients. One treated patient cannot be included because she was completely lost to follow-up. Twenty-nine patients were found, on review (K.A.K.), to be ineligible (17 treated and 12 control) and are excluded since they were not followed up. Patients were excluded after randomisation for the following reasons; advanced disease (six treated, three control), >65 years of age (four treated, one control), abnormal liver function tests (one treated, seven control), intercurrent disease (three treated), node negative (two treated, one control) and lymphoma (one treated). The characteristics of the patients eligible for analysis of relapse and toxicity are given in Table I, which shows that there are no major imbalances of prognostic factors between the treated and control groups.

*Relapse-free survival*

Adjuvant chemotherapy significantly prolonged relapse free survival (Figure 1, Table II). After stratification for menopausal status and tumour size, $\chi^2_1$ is 11.9 ($P = 0.0006$). Controlling for menopausal status, tumour size, age, tumour grade or receptor status does not substantially alter this treatment effect.

The percentage relapse free at 5 years is improved by treatment from 27% to 38% (with confidence intervals of 21–33% and 32–44% respectively) and represents a relative improvement (RI) of 42% ((38 − 27)/27) in relapse rate or a 30% reduction in the odds of relapse (OR). The median prolongation of relapse free survival is 14 months, from 628 days to 1,057 days (with confidence intervals of 554–808 days and 836–1,290 days respectively).

There were 181 (66%) patients who recurred in the treated group and 199 (76%) in the control group. There was no significant difference in the distribution of metastases between the treatment and control groups ($\chi^2_1 = 0.18$; $P = 0.67$; Table III).

**Table I** Patient characteristics

| | *Treated (277 patients)* | | *Control (263 patients)* | |
|---|---|---|---|---|
| | *n* | *%* | *n* | *%* |
| Age | | | | |
| Less than 50 | 116 | 42 | 115 | 44 |
| 50 plus | 161 | 58 | 148 | 56 |
| Menopausal status | | | | |
| Premenopausal | 118 | 43 | 115 | 44 |
| Post-menopausal | 130 | 47 | 117 | 44 |
| Hysterectomy | 29 | 10 | 31 | 12 |
| Tumour size | | | | |
| <2.0 cm | 27 | 10 | 18 | 7 |
| 2.0–4.9 cm | 162 | 58 | 170 | 65 |
| ⩾5.0 cm | 88 | 32 | 74 | 28 |
| Not known | – | – | 1 | <1 |
| Histology | | | | |
| Infiltrating duct | 221 | 80 | 222 | 84 |
| Infiltrating lobular | 33 | 12 | 29 | 11 |
| Other | 11 | 4 | 4 | 2 |
| Not reviewed | 12 | 4 | 8 | 3 |
| Grade | | | | |
| I | 29 | 11 | 27 | 10 |
| II | 153 | 55 | 156 | 59 |
| III | 81 | 29 | 68 | 26 |
| Not reviewed | 14 | 5 | 12 | 5 |
| Oestrogen receptors | | | | |
| Negative | 49 | 18 | 58 | 22 |
| Positive | 130 | 47 | 137 | 52 |
| Not assayed | 98 | 35 | 68 | 26 |
| Progesterone receptors | | | | |
| Negative | 54 | 19 | 65 | 25 |
| Positive | 88 | 32 | 90 | 34 |
| Not assayed | 135 | 49 | 108 | 41 |

| At risk | | | | | | | | | | | |
|---|---|---|---|---|---|---|---|---|---|---|---|
| AVCMF | 276 | 225 | 165 | 130 | 104 | 88 | 61 | 41 | 34 | 10 | 10 |
| Control | 263 | 182 | 123 | 97 | 71 | 55 | 34 | 26 | 15 | 15 | 15 |

**Figure 1** Relapse-free survival for all eligible patients. AVCMF (181/276 relapsed; control (199/263 relapsed); $\chi^2_1 = 11.68$; $P = 0.006$.

Prolongation of relapse-free survival was highly significant in premenopausal patients ($\chi^2_1 = 8.8$; $P = 0.003$: Figure 2a) but of only borderline significance in post-menopausal patients ($\chi^2_1 = 3.3$; $P = 0.07$: Figure 2b). The relative improvement in 5-year relapse rate was 73% in the premenopausal group (OR = 37%) and 17% (OR = 22%) in the post-menopausal group. The median prolongation of relapse free survival was 17 months (from 594 to 1,112 days) in the premenopausal group and 8 months (from 741 to 978 days) in the post-menopausal group.

*Survival*

There was no significant effect of chemotherapy on overall survival (Figure 3), with an RI of 6% and an OR of 9%. After stratification for menopausal status and tumour size, $\chi^2_1 = 1.27$; $P = 0.26$. There was no effect of treatment on survival in any sub-group (Table II). Controlling for menopausal status, tumour size, age, tumour grade and receptor status does not alter this result.

*Steroid hormone receptors*

Measurements of oestrogen receptor (ER) were performed on 69% (374/540) of tumours and of progesterone receptor (PR) on 55% (297/540) of tumours (Table I). Seventy-one per cent of tumours were ER positive and 60% PR positive ($\geqslant 5$ fmol mg$^{-1}$ cytosol protein). There were no significant differences in patient characteristics between those with and without receptor measurements. When all patients were analysed, there was significant prolongation of relapse-free survival in patients with ER positive (RI = 44%; OR = 28%) and ER negative tumours (RI = 47%; OR = 37%) (Table II). There was a highly significant prolongation of relapse-free survival in patients with PR positive tumours (RI = 68%; OR = 41%; $\chi^2_1 = 7.8$; $P = 0.005$); the effect in patients with PR negative tumours just failed to reach significance, (RI = 68%; OR = 32% $\chi^2_1 = 3.4$; $P = 0.06$), but the numbers in this group were smaller. There was no relationship between receptor status and overall survival for any group (Table II).

Relapse-free survival and receptor results in relation to the menopause are shown in Table IV. The numbers in these subgroups are small and the results must be viewed with caution. The greatest relative improvement of 117% (OR = 48%), accompanied by the longest absolute prolongation of median relapse-free survival was in the premenopausal PR positive subgroup (treated group 55 months *vs* control group 19 months; $\chi^2_1 = 6.2$; $P = 0.01$). In the post-menopausal group, treated patients with PR negative tumours fared better, although this result failed to reach formal levels of significance. None of the subgroups analysed had a significantly prolonged survival in treated patients.

*Histological grade*

Histological grade was assessed on the tumours from all but 14 of the treated patients and all but 12 of the controls. Of those assessed, 11% were grade I, 60% were grade II and 29% were grade III. There was no difference in the distribution of grades between the treated and control groups (Table I). Chemotherapy reduced the risk of relapse in all grades;

**Table II** Effect of treatment in subgroups of patients

| | Relapse-free survival | | | | | | | | Overall survival | | | | | | | |
|---|---|---|---|---|---|---|---|---|---|---|---|---|---|---|---|---|
| | Treated | | Control | | | | | | Treated | | Control | | | | | |
| | *N* | *R* | *N* | *R* | $\chi^2_1$ | *P* | *RI* | *OR* | *N* | *D* | *N* | *D* | $\chi^2_1$ | *P* | *RI* | *OR* |
| Overall | 276 | 181 | 263 | 199 | 11.7 | <0.001 | 42 | 30 | 294 | 143 | 275 | 140 | 0.6 | 0.45 | 6 | 9 |
| Menopausal status | | | | | | | | | | | | | | | | |
| Pre | 128 | 78 | 126 | 95 | 8.8 | 0.003 | 73 | 37 | 131 | 59 | 130 | 64 | 0.5 | 0.49 | 8 | 12 |
| Post | 148 | 103 | 137 | 104 | 3.3 | 0.07 | 17 | 22 | 159 | 80 | 142 | 75 | 0.5 | 0.46 | 8 | 11 |
| Age | | | | | | | | | | | | | | | | |
| <50 | 116 | 71 | 115 | 88 | 8.1 | 0.004 | 69 | 37 | 119 | 54 | 119 | 61 | 0.8 | 0.37 | 12 | 16 |
| ⩾50 | 160 | 110 | 148 | 111 | 4.2 | 0.04 | 27 | 24 | 175 | 89 | 156 | 79 | 0.1 | 0.83 | 1 | 3 |
| Tumour size | | | | | | | | | | | | | | | | |
| < 5 cm | 189 | 118 | 188 | 138 | 9.3 | 0.002 | 42 | 32 | 195 | 85 | 194 | 94 | 1.5 | 0.23 | 12 | 17 |
| ⩾ 5 cm | 87 | 63 | 74 | 60 | 3.0 | 0.08 | 45 | 27 | 92 | 53 | 77 | 44 | 0.0 | 0.94 | 0 | 2 |
| ER status | | | | | | | | | | | | | | | | |
| Positive | 130 | 84 | 137 | 102 | 4.8 | 0.03 | 44 | 28 | 130 | 54 | 137 | 61 | 0.3 | 0.60 | 6 | 9 |
| Negative | 49 | 35 | 58 | 50 | 4.4 | 0.04 | 47 | 37 | 49 | 36 | 58 | 40 | 0.0 | 0.88 | −14 | −4 |
| PR status | | | | | | | | | | | | | | | | |
| Positive | 88 | 50 | 90 | 67 | 7.8 | 0.005 | 68 | 41 | 88 | 32 | 90 | 38 | 0.7 | 0.40 | 9 | 18 |
| Negative | 54 | 38 | 65 | 52 | 3.4 | 0.06 | 68 | 32 | 54 | 31 | 65 | 38 | 0.1 | 0.80 | 10 | 6 |
| Grade | | | | | | | | | | | | | | | | |
| I | 29 | 10 | 27 | 18 | 7.3 | 0.007 | 74 | 65 | 29 | 6 | 27 | 8 | 0.83 | 0.36 | 5 | 39 |
| II | 153 | 106 | 156 | 121 | 4.4 | 0.04 | 37 | 24 | 153 | 71 | 156 | 74 | 0.09 | 0.77 | 6 | 5 |
| III | 80 | 55 | 68 | 53 | 3.5 | 0.06 | 46 | 31 | 81 | 48 | 68 | 45 | 1.0 | 0.33 | 10 | 18 |

N, number of patients in subgroup; R, number of patients who have relapsed; D, number of patients who have died; RR, relative risk reduction in treated group; OR, odds reduction in treated group.

**Table III** Sites of first recurrence

| | Treated (181) | Control (199) |
|---|---|---|
| | n (%[a]) | n (%[a]) |
| Local and regional recurrence only | 87 (49) | 92 (47) |
| Distant recurrence | 91 (51) | 105 (53) |
| Site of distant recurrences | | |
| Bone | 36 (20) | 46 (23) |
| Liver | 9 (5) | 8 (4) |
| Lung | 26 (15) | 25 (13) |
| Other[b] | 13 (7) | 12 (6) |
| Distant and local recurrence | 7 (4) | 14 (7) |
| Site not reviewed[c] | 3 | 2 |

[a]% of those reviewed. [b]Ascites (2C), brain (5T, 2C), contralateral breast (6T, 5C), contralateral axilla (1T, 2C), contralateral nodes and axilla (1T, 1C). [c]Died with disease, recurrence not recorded (2T, 1C), notes lost (1T, 1C).

this effect appeared greater in patients with grade I tumours (RI = 74%; OR = 65%) than in those with grade II (RI = 37%; OR = 24%) or grade III (RI = 46%; OR = 31%; Table II). The relationship between grade and relapse-free survival was analysed according to menopausal status (Table V). Because of the relatively low numbers of grade I tumours, grades I and II were combined. The greatest prolongation of relapse-free survival was seen in premenopausal women with grade I/II tumours (median 63 months *vs* 24 months; RI = 97%; OR = 43%; $P = 0.004$). The effect of AVCMF on relapse-free survival was not significant for premenopausal women with grade III tumours or in either post-menopausal group.

### Chemotherapy

Eighty per cent of eligible patients in the treatment group received eight cycles of treatment, 88% seven cycles or more and 91% four cycles or more; 3% of eligible patients ran-

**Figure 2** Relapse-free survival by menopausal status for eligible patients. a, Premenopausal patients: AVCMF (78/128 relapsed); control (95/126 relapsed); $\chi^2_1 = 8.75$; $P = 0.003$. b, Post-menopausal patients: AVCMF (103/148 relapsed; control (104/137 relapsed); $\chi^2_1 = 3.26$; $P = 0.07$.

**Figure 3** Survival for all randomised patients. AVCMF (144/294 died); control (140/275 died); $\chi^2_1 = 0.50$; $P = 0.48$.

domised to treatment received no chemotherapy. The reasons for not receiving the full course of eight cycles of chemotherapy were toxicity (22%), intercurrent illness not associated with treatment (15%), patient refusal (37%) and administrative errors mainly involving failure to give the eighth cycle (26%).

### Toxicity

The proportion of patients affected and the number of cycles in which toxic effects were seen are summarised in Table VI. Severe leukopenia and thrombocytopenia were rare. Most patients had nausea and vomiting on at least one occasion. When these symptoms were analysed on a per cycle basis, 66% of cycles were associated with nausea and 47% with vomiting. Treatment was delayed by one week in 74 cycles and longer in 39. Alopecia was virtually universal but other side-effects such as stomatitis or neuropathy were uncom-

**Table IV** Effect of treatment on RFI by menopausal and receptor status

| | *N* | *R* | *Median RFI* | *% RF at 5 years* | *RI* | *OR* | $\chi^2_1$ | *P* |
|---|---|---|---|---|---|---|---|---|
| Premenopausal ER positive | | | | | | | | |
| Treated | 58 | 34 | 1092 | 42 | 63 | 32 | 2.8 | 0.10 |
| Control | 54 | 39 | 771 | 26 | | | | |
| Premenopausal ER negative | | | | | | | | |
| Treated | 28 | 20 | 645 | 35 | 77 | 42 | 3.4 | 0.07 |
| Control | 30 | 27 | 411 | 20 | | | | |
| Premenopausal PR positive | | | | | | | | |
| Treated | 55 | 31 | 1677 | 49 | 117 | 48 | 6.2 | 0.01 |
| Control | 43 | 33 | 582 | 22 | | | | |
| Premenopausal PR negative | | | | | | | | |
| Treated | 16 | 12 | 467 | 21 | 12 | 10 | 0.1 | 0.77 |
| Control | 25 | 21 | 411 | 19 | | | | |
| Post-menopausal ER positive | | | | | | | | |
| Treated | 72 | 50 | 1158 | 36 | 28 | 22 | 1.8 | 0.18 |
| Control | 83 | 63 | 619 | 28 | | | | |
| Post-menopausal ER negative | | | | | | | | |
| Treated | 21 | 15 | 734 | 29 | 1 | 27 | 0.9 | 0.34 |
| Control | 28 | 23 | 554 | 28 | | | | |
| Post-menopausal PR positive | | | | | | | | |
| Treated | 33 | 19 | 1155 | 37 | 19 | 31 | 1.8 | 0.18 |
| Control | 47 | 34 | 741 | 31 | | | | |
| Post-menopausal PR negative | | | | | | | | |
| Treated | 38 | 26 | 946 | 41 | 56 | 39 | 3.3 | 0.07 |
| Control | 40 | 31 | 497 | 26 | | | | |

N, number of patients in subgroup; R, number of patients who have relapsed; RR, relative risk reduction in treated group; OR, odds reduction in treated groups.

**Table V** Effect of treatment on RFI by menopausal status and grade

| | *N* | *R* | *Median RFI* | *% RF at 5 years* | *RR* | $\chi^2_1$ | *P* |
|---|---|---|---|---|---|---|---|
| Grade I/II | | | | | | | |
| Treated | 182 | 116 | 1238 | 41 | 46 | 8.7 | 0.003 |
| Control | 183 | 139 | 741 | 28 | | | |
| Grade III | | | | | | | |
| Treated | 80 | 55 | 775 | 33 | 46 | 3.5 | 0.06 |
| Control | 68 | 53 | 482 | 23 | | | |
| Premenopausal grade I/II | | | | | | | |
| Treated | 77 | 43 | 1915 | 52 | 97 | 8.5 | 0.004 |
| Control | 86 | 65 | 724 | 26 | | | |
| Premenopausal grade III | | | | | | | |
| Treated | 42 | 29 | 638 | 32 | 74 | 2.3 | 0.13 |
| Control | 32 | 26 | 482 | 18 | | | |
| Post-menopausal grade I/II | | | | | | | |
| Treated | 105 | 73 | 987 | 33 | 16 | 1.7 | 0.19 |
| Control | 97 | 74 | 741 | 29 | | | |
| Post-menopausal grade III | | | | | | | |
| Treated | 38 | 26 | 887 | 35 | 32 | 1.3 | 0.25 |
| Control | 36 | 27 | 456 | 27 | | | |

N, number of patients in subgroup; R, number of patients who have relapsed; RR, relative risk reduction in treated group.

mon. In particular, there were no episodes of cardiac failure.

The severity and duration of nausea and vomiting are outlined in Figure 4. Severe symptoms were uncommon. However, when symptoms occurred, they lasted for longer than 24 h in approximately half the cycles assessed. An assessment of the 'quality of life' of patients is shown in Figure 4. Patients were asked how long they were unwell, how long they were unable to go to work (or perform housework) and how long they were confined to bed if they had toxicity. Patients were unwell in 58% of cycles. In 37% of cycles patients took time off work and in 30% they were confined to bed for a variable period.

*Second primary tumours*

Second primary tumours occurred in two of the control (cervix, rectum) and three of the treated group (cervix, lung, oesophagus). No leukaemias have been recorded.

**Table VI** Toxicity attributed to chemotherapy

| | Patients[a] | | Cycles[b] | |
|---|---|---|---|---|
| *Haematology* | *n (253)* | *%* | *n (1651)* | *%* |
| Hb (g $dl^{-1}$) | | | | |
| 9.5–10.9 | 28 | 11 | 75 | 5 |
| <9.5 | 6 | 2 | 6 | <1 |
| WBC count ($\times 10^9 l^{-1}$) | | | | |
| 3–3.9 | 99 | 39 | 233 | 14 |
| 2–2.9 | 5 | 2 | 5 | <1 |
| <2 | 1 | < 1 | 1 | <1 |
| Platelets ($\times 10^9 l^{-1}$) | | | | |
| 70–99 | 2 | < 1 | 2 | <1 |
| <70 | 1 | < 1 | 1 | <1 |
| *Side-effects* | *n (251)* | *%* | *n (1845)* | *%* |
| Nausea | 238 | 95 | 1224 | 66 |
| Vomiting | 210 | 84 | 875 | 47 |
| Rash | 36 | 14 | 60 | 3 |
| Stomatitis | 62 | 25 | 91 | 5 |
| Diarrhoea | 63 | 25 | 130 | 7 |
| Neuropathy | 60 | 24 | 95 | 5 |
| Hair loss | 244 | 97 | – | – |
| Cardiac failure | 0 | 0 | – | – |

[a]Recorded if the toxic event occurred on at least one occasion on at least one occasion per patient. [b]Number of treatment cycles in which the toxic event occurred.

### Discussion

This study shows that administration of eight cycles of AVCMF after mastectomy to women with axillary node involvement delays local and distant relapse by a median of 14 months. This difference in relapse-free survival has not been translated into an equivalent advantage in overall survival. It was hoped that the addition of adriamycin and vincristine to CMF would enhance the therapeutic effect; our results do not show a greater effect at this stage of follow-up compared with CMF trials. At a median follow-up of 7 years, 50% of patients have died. These deaths are in women who presumably had undetected but extensive micrometastatic disease present at mastectomy and who would therefore be expected to recur early after surgery. It remains to be seen whether the more intensive chemotherapy reported here will affect survival in patients presumed to have a small micrometastatic burden at mastectomy. This group would be expected to have a later recurrence and death and possibly be the very patients to benefit from adjuvant chemotherapy.

In this study not all patients randomised to treatment received chemotherapy; eleven ineligible patients (3.7%) may not have had chemotherapy and eight patients (2.7%), although eligible, did not receive any treatment, usually due to patient refusal. Analysis of survival with or without these groups does not alter the result.

The median prolongation of the time to relapse was 14

**Figure 4** Side-effects of treatment: nausea and vomiting and aspects of quality of life.

months for all patients: 17 months in the premenopausal group and 8 months in the post-menopausal group. It is clearly difficult to make judgements concerning the trade off in cost related to the toxicity and inconvenience of chemotherapy and benefit related to the increased period free from recurrence of disease. The treatment was relatively myelosuppressive but there were no infective or other toxic deaths. There were no episodes of cardiac failure, although the total dose of adriamycin (400 mg) was well below that which can produce cardiotoxicity and the trial was confined to patients below age 65 because of this potential risk. Just over half of the cycles of chemotherapy were associated with symptoms of toxicity and in half of these, the symptoms lasted for more than 24 h. Thus, in chemotherapy term, this regimen of treatment is relatively non-toxic.

*The significance of AVCMF in relation to other trials*

The results of our trial must be set in the context of the Early Breast Cancer Trialists' Collaborative Group (1988) 'overview' of all trials of combination chemotherapy with a no treatment control arm. In the overview, trials of combination chemotherapy were divided into three groups: CMF regimens, CMF regimens with extra cytotoxic agents and regimens without some or all of C, M, F.

The trial reported here falls into the group of CMF regimens with extra cytotoxic agents. It is the only study in the group with more than 150 patients randomised, where the data are available and where there is adequate follow-up. Patients from this trial represent 39% (569/1,467) of those analysed in this group. The results from this trial are consistent with the overview for this group which shows a highly significant improvement in relapse-free survival and a clear survival advantage in young women, but no clear survival advantage in older women. The overview shows a reduction in the odds of relapse of 30% both for this group and for all polychemotherapy trials; in this analysis the odds reduction is also 30%. The reduction in the odds of death of 26% in young women shown for our trial in the overview is similar to the average reduction of 20% for all trials in this group and the same as the overall average for all polychemotherapy trials of 26%. With a median of 7 years follow-up the odds reduction is now 16%.

This regimen, with a reduction in the odds of death of 26% in the overview, appears to be intermediate in effectiveness between CMF (OR = 37%) and other multiple agents (OR = 12%), and is very close to the overall average improvement of 26% reported for all polychemotherapy trials and 22% for all chemotherapy trials. Care must be taken when comparing groups of chemotherapies. The overview shows no significant heterogeneity between the chemotherapy trials reported, so that such comparisons between groups of chemotherapies must be regarded as useful for the generation of hypotheses, rather than being definitive.

*Steroid hormone receptors*

ER and PR were measured on 69% and 55% of tumours. Analysis showed no significant relationship between ER and relapse-free survival and overall survival whereas there was a significant effect of chemotherapy upon relapse-free survival but not overall survival in patients with PR positive tumours (Table II). This appeared to be mainly in the premenopausal group (Table IV) but the numbers in the subsets were small and must be viewed with caution. However, although not reliable in its own right, the result is consistent with most other trials where this has been examined since most show a significant advantage for treatment for relapse-free survival and overall survival in premenopausal women with receptor positive tumours (Table VII). Most trials show a positive association between the effectiveness of chemotherapy and the induction of amenorrhoea. Taken together the relationship between ovarian suppression and receptors with the effect of chemotherapy suggests that it is acting, in part, by an indirect endocrine effect.

**Table VII** Effect of chemotherapy in relation to induced amenorrhoea and steroid receptors

| *Trial* | | *Amenorrhoea* | *Receptors* |
|---|---|---|---|
| *Relapse-free survival* | | | |
| Milan (Bonadonna *et al.*, 1985) | CMF | NS | – |
| Guys/Manchester (Howell *et al.*, 1984; Padmanabhan *et al.*, 1986) | CMF | + | + (PR) |
| Denmark (Brinckler *et al.*, 1987) | C | + | + (ER) |
| | CMF | NS | NS (ER) |
| Leiden (Repelaer van Driel *et al.*, 1986) | CMF | NS | – |
| Nijmegan (Beex *et al.*, 1988) | CMF | + | + (PR) |
| Ludwig (Ludwig Breast Cancer Study Group, 1985) | CNF±p | + | + (ER) |
| ECOG (Tormey, 1984) | CMF±P | + | + (ER) |
| West Midlands | AVCMF | – | + (PR) |
| *Survival* | | | |
| Milan | CMF | NS | – |
| Guys/Manchester | CMF | + | + (PR) |
| Nijmegan | CMF | + | + (PR) |
| ECOG | CMF±p | + | + (PR) |
| West Midlands | AVCMF | – | NS (PR) |

NS, not significant; +, positive effect; –, not reported

*Histological grade*

We have shown that AVCMF is most active in prolonging relapse-free survival in premenopausal women with grade I and II tumours. These tumours are more likely to be steroid hormone receptor positive compared with grade III tumours. This supports the association in this study between PR positive tumours and prolonged relapse free survival and is consistent with a partial endocrine effect of chemotherapy. However, there was also an effect in grade III tumours overall but this failed to reach conventional significance levels ($P = 0.06$). These results are not consistent with other studies. Fisher *et al.* (1986) reported that melphalan was active only in women with grade III tumours. Brinckler *et al.* (1987) showed that CMF was equally active in women with grade II and grade III tumours, but was inactive in women with grade I tumours. It is not clear why the results of the three studies should be as divergent: it is likely that it is an effect of small numbers and the subjectiveness of grading. The latter was standardised in this report by having a single pathologist look at all the slides.

*Conclusion*

Taken in isolation the results presented here would not give a clear indication for the general use of adjuvant chemotherapy in node positive breast cancer. A useful prolongation of relapse-free survival was seen in premenopausal women, but that seen in post-menopausal women was approximately equivalent to the duration of treatment with chemotherapy after surgery. It will be important to determine whether this high risk group of young women fare better when given adjuvant chemotherapy compared with adjuvant endocrine therapy: there is clearly a need for clinical trials to compare the effects of each treatment. However, greater improvements in survival will only come with improved chemotherapy or the introduction of entirely new treatments. The effectiveness of adjuvant chemotherapy might be improved by increasing the intensity of treatment, possibly with haemopoietic growth factors, by giving treatment early, possibly even before surgery and by developing new agents. It is in our view preferable to examine these important factors in well planned clinical trials than to recommend prematurely the use of adjuvant chemotherapy in routine clinical practice, particularly in post-menopausal women.

Participating Surgeons and Hospitals: Birmingham General Hospital: J. Alexander-Williams, R.M. Baddeley, N.J. Dorricot, M.R.B. Keighley, G.D. Oates; Bromsgrove General Hospital: J.H. Burman, G.F. Grave; Burton District Hospital and Burton General Hospital: H.C. De Castella, S. Glick; Dudley Road Hospital, Birmingham: P.G. Bevan, I.A. Donovan, M. Obeid; Edgbaston Nursing Home, Birmingham; George Eliot Hospital, Nuneaton: J.R. Moffat; Good Hope District General Hospital, Sutton Coldfield: W.M. Lein, R.S. Rihan, D.R. Thomas; Kidderminster General Hospital: P.R. Armistead, R.E. Gibbins, E.W. Gillison; Longton Cottage Hospital; North Staffordshire Royal Infirmary, Stoke-on-Trent: L.J. Lawson, E.R. Monypenny; Queen Elizabeth Hospital, Birmingham: J. Fielding, J.D. Hammer, J.G. Temple; Sandwell District Hospital, West Bromwich: J.D. Hennessy; Selly Oak Hospital, Birmingham: J.P. Grant, A.R. Leask, J. Morrison, N.E. Winstone; Solihull Hospital: R.W. Tudor; St Chad's Hospital, Birmingham; Hospital of St Cross, Rugby: T.A. Waterworth; Stratford-upon-Avon Hospital: R.T. Marcus; Victoria Hospital, Lichfield: F.R. Hurford; Walsall General Hospital & Manor Hospital, Walsall: K.D. Fortes-Mayer; Walsgrave Hospital: G.A. Court, R.W. Parker; Warneford General Hospital, Leamington Spa: M.D. Lord; Warwick General Hospital: J.D. Marsh; Worcester Royal Infirmary: H.T. Williams; Wordsley Hospital, Stourbridge: H. Kramer.

We wish to thank the Cancer Research Campaign for grants to the project, Eli Lilly, Farmitalia Carlo Erba and Lederle for their financial assistance and also the Lions Club International District 105 and the many other groups and individuals within the West Midlands who provided support by fund raising activities or donation. In particular, we thank Lady Veronica Booth for working so hard as patron of our local fund raising campaign. Members of the WMOA Breast Cancer Study Group were K. Arthur, A. Banks, W. Bond, D. Cove, I. Donovan, C. Fortes-Meyer, J. Harnden, A. Howell, E.R. Monypenny, J.M. Morrison, C. Newman, G.D. Oates, A. Rowse, R. Walker, J.A.H. Waterhouse and K. Woods. The regimen of chemotherapy was developed by Dr L. Price and Dr B. Hill. Mr Alan Hughes and Miss Sharon Hughes carried out the receptor estimation. We are most grateful to Miss Sally Burman, Mrs Jane Gaines, Mr Alan Marson, Dr Abe Minawa, Mrs Linda Pitt, Mrs Linda Ward and Ms Wendy Gillespie for their help in preparing the data. We thank all the staff of the West Midlands Regional Cancer Registry and West Midlands Cancer Research Campaign Clinical Trials Unit who have given assistance. The co-operation of participating surgeons, pathologists, radiologists and medical physicists throughout the West Midlands has been crucial to the success of the trial and we wish to acknowledge their continuing support. We also wish to thank Dr T. Powles for his hard work in carrying out the trial audit.

### References

BEEX, L.V., MACKENZIE, M.A., RAEMAEKERS, J.M. & 4 others (1988). Adjuvant chemotherapy in premenopausal patients with primary breast cancer; relation to drug induced amenorrhoea, age and the progesterone receptor status of the tumour. *Eur. J. Clin. Oncol.*, **24**, 719.

BLOOM, H.J.G. & RICHARDSON, W.W. (1957). Histological grading and prognosis in breast cancer. *Br. J. Cancer*, **11**, 359.

BONADONNA, G., BRUSAMOLINO, E., VALAGUSSA, P. *et al.* (1976). Combination chemotherapy as an adjuvant treatment in operable breast cancer. *N. Engl. J. Med.*, **294**, 405.

BONADONNA, G., VALAGUSSA, P., ROSSI A. & 4 others (1985). Ten year experience with CMF-based adjuvant chemotherapy in resectable breast cancer. *Breast Cancer Res. Treat.*, **5**, 95.

BRINCKLER, H., ROSE, C., RANK, F. & 5 others (1987). Evidence of a castration-mediated effect of adjuvant cytotoxic chemotherapy in premenopausal breast cancer. *J. Clin. Oncol.*, **5**, 1771.

COX, D.R. (1972). Regression models and life tables. *J. R. Stat. Soc. Series B*, **34**, 187.

Early Breast Cancer Trialists Collaborative Group (1988). Effects of adjuvant tamoxifen and of cytotoxic therapy on mortality in early breast cancer: an overview of 61 randomised trials among 28,896 women. *N. Engl. J. Med.*, **319**, 1681.

ELSTON, C.W., GRESHAM, T.A., RAO, G.S. *et al.* (1982). The Cancer Research Campaign (King's/Cambridge) trial for early breast cancer: clinico-pathological aspects. *Br. J. Cancer*, **45**, 655.

FISHER, B., FISHER, E.R., REDMOND, C. and participating NSAPB investigators (1986). Ten year results from the national surgical adjuvant breast and bowel project (NSAPB) clinical trial evaluating the use of L-phenylalanine mustard (L-PAM) in the management of primary breast cancer. *J. Clin. Oncol.*, **4**, 929.

FORREST, A.P.M., ROBERTS, M., CANT, E. & SHIVAS, A.A. (1976). Simple mastectomy and pectoral node biopsy. *Br. J. Surg.*, **63**, 569.

GOLDIE, J.H. & COLDMAN, A.J. (1979). A mathematical model for relating the drug sensitivity of tumours to their spontaneous mutation rate. *Cancer Treat. Rep.*, **63**, 172.

HOWELL, A., GEORGE, W.D., CROWTHER, D. & 7 others (1984). Controlled trial of adjuvant chemotherapy with cyclophosphamide, methotrexate and fluorouracil for breast cancer. *Lancet*, **ii**, 307.

KAPLAN, E.L. & MEIER, P. (1958). Nonparametric estimation from incomplete observations. *J. Am. Stat. Assoc.*, **53**, 457.

LUDWIG BREAST CANCER STUDY GROUP (1985). Adjuvant chemotherapy (CMF) with or without low dose prednisone (p) in premenopausal patients with metastases in 1–3 axillary lymph nodes. Ludwig trial 1 (LBCS1). *Proc. Am. Soc. Clin. Oncol.*, **4**, 53.

MCGUIRE, W.L. & DE LA GARZA, M. (1973). Improved sensitivity in the measurement of estrogen receptor in human breast cancer. *J. Clin. Endocrinol. Metab.*, **37**, 986.

MORRISON, J.M., HOWELL, A., GRIEVE, R.J. & 4 others (1984). West Midlands Oncology Association trials of adjuvant chemotherapy for operable breast cancer. In *Adjuvant Therapy of Cancer IV*, Jones, S.E. & Salmon, S.E. (eds) p. 253. Grune & Stratton: New York.

MORRISON, J.M., HOWELL, A., GRIEVE, R.J., MONYPENNY, I.J., KELLY, K.A. & WATERHOUSE, J.A. (1987). West Midlands Oncology Association trials of adjuvant chemotherapy for operable breast cancer. In *Adjuvant Therapy of Cancer V*, Salmon, S.E. (ed) p. 311. Grune & Stratton: Orlando, Fla.

MORRISON, J.M., HOWELL, A., GRIEVE, R.J., MONYPENNY, I.J., MINAWA, A. & WATERHOUSE, J.A. (1981). The West Midlands Oncology Association trials of adjuvant chemotherapy for operable breast cancer. In *Adjuvant Therapy of Cancer III*, Salmon, S.E. & Jones, S.E. (eds) p. 403. Grune & Stratton: New York.

NISSEN-MEYER, R., KJELLGREN, K., MALMIO, K., MANSSON, B. & NORIN, T. (1978). Surgical adjuvant chemotherapy. Results with one short course of cyclophosphamide after mastectomy for breast cancer. *Cancer*, **41**, 2088.

PADMANABHAN, N., HOWELL, A. & RUBENS, R.D. (1986). Mechanisms of action of adjuvant chemotherapy in early breast cancer. *Lancet*, **ii**, 411.

PETO, R., PIKE, M.C., ARMITAGE, P. & 7 others (1977). Design and analysis of randomised clinical trials requiring prolonged observation of each patient: part II, analysis and examples. *Br. J. Cancer*, **35**, 1.

PRICE, L.A., HILL, B.T., MARKS, P., HOWELL, A., MONYPENNY, I. & MORRISON, J.M. (1983). Twenty four hour combination chemotherapy: a feasibility study with implications for improved adjuvant treatment of breast cancer. *Eur. J. Cancer Clin. Oncol.*, **19**, 1.

REPELAER VAN DRIAL, O.J., WELVAART, K., VAN DE VELDE, C.J. & ZWAVELING, A. (1987). Relation of overall survival/relapse free survival and axillary nodal status in patients with primary breast cancer, treated with an adjuvant low-dose CMF-regimen (EORTC 09771). ECCO-4. Fourth European Conference on Clinical Oncology and Cancer Nursing, p. 114.

SKIPPER, H.E. (1978). Adjuvant chemotherapy. *Cancer*, **41**, 936.

TORMEY, C. (1984). Adjuvant systemic therapy in postoperative node positive patients with breast carcinoma: the CALGB trial and the ECOG premenopausal trial. In *Recent Results in Cancer Research. Adjuvant Chemotherapy of Breast Cancer*, p. 155. Springer-Verlag: Heidelberg.